# Real-world first-line serplulimab therapy for advanced esophageal cancer: effectiveness, safety, and clinical implications

**DOI:** 10.3389/fmed.2025.1637458

**Published:** 2025-08-06

**Authors:** Fei Yan, Jingni Zhu, Qibing Wu, Yuanyuan Ren, Xinnian Yu, Zijian Sun, Yang Liu, Changjiang Sun, Yan Sun

**Affiliations:** ^1^Department of Medical Oncology, Jiangsu Cancer Hospital & Jiangsu Institute of Cancer Research & The Affiliated Cancer Hospital of Nanjing Medical University, Nanjing, China; ^2^Department of Radiation Oncology, The First Affiliated Hospital of Anhui Medical University, Hefei, China; ^3^Department of Medical Oncology, Taizhou People’s Hospital, Taizhou, China; ^4^Department of Medical Oncology, Sichuan Cancer Hospital and Institute, Chengdu, China; ^5^Department of Medical Oncology, Subei People’s Hospital of Jiangsu Province, Yangzhou, China

**Keywords:** esophageal cancer, advanced cancer, serplulimab, treatment efficacy, safety, real-world evidence

## Abstract

**Objective:**

The phase III ASTRUM-007 trial demonstrated significant clinical benefit in patients with PD-L1-positive esophageal squamous cell carcinoma (ESCC) treated with first-line serplulimab plus chemotherapy. This multicenter, observational cohort study aimed to evaluate the real-world outcomes, and address evidence gaps in broader patient populations.

**Methods:**

This multicenter real-world cohort study collected the data of patients with locally advanced or metastatic esophageal cancer who received first-line serplulimab treatment, regardless of histologic type and PD-L1 expression. The outcomes included real-world progression-free survival (rwPFS), objective response rate (ORR), disease control rate (DCR), overall survival (OS) and safety.

**Results:**

Among 104 included patients, the median number of serplulimab treatment cycles was four; 10 patients (9.6%) concurrently received targeted therapy, 21 (20.2%) received radiotherapy, and 97 received chemotherapy (93.3%). The confirmed ORR was 40.0% (95% confidence interval [CI]: 29.8–50.9%) and the DCR was 97.8% (95% CI: 92.2–99.7%). With a median follow-up time of 6.8 months, the median rwPFS was 12.00 months (95% CI: 8.87-not reached [NR]). The median OS was not reached (95% CI: 13.27-NR), with a 1-year OS rate of 73.5% (95% CI: 60.4–89.3%). Subgroup analysis showed prolonged median PFS in patients aged ≥65 years than those <65 years (12.00 vs. 6.87 months, *p* = 0.022). Only two serious adverse events were reported (one hyperkalemia and one decreased white blood cell count).

**Conclusion:**

This real-world study supports the effectiveness and safety of serplulimab-based regimens as a first-line treatment for patients with locally advanced or metastatic esophageal cancer, regardless of their diverse characteristics.

## 1 Introduction

Esophageal cancer remains a major global health challenge, with poor prognosis and limited treatment options for advanced-stage disease. In 2022, it was the 11th most commonly diagnosed malignancy and the seventh leading cause of cancer-related deaths worldwide, with an estimated 510,716 new cases (2.6% of all cancers) and 445,129 deaths (4.6% of all cancer-related deaths) (1). In particular, China bears a disproportionate burden of esophageal cancer, accounting for 53.7% of new cases and 39.2% of global mortality (2); while in the United States, the 2024 SEER data projects 22,370 new cases and 16,130 deaths (3). Due to the insidious and nonspecific nature of early swallowing difficulty in esophageal cancer, the majority of patients in China present with locally advanced or metastatic disease at diagnosis, when surgical resection is no longer an option (2). Furthermore, even those diagnosed at an early stage face a high risk of recurrence despite radical surgery or definitive radiochemotherapy (4).

The systemic treatment landscape for solid tumors has undergone revolutionary changes since 1970, driven by endocrine therapy, targeted therapy, and immunotherapy (5). Particularly, the integration of immune checkpoint inhibitors (ICIs) into the treatment landscape of esophageal cancer has significantly improved clinical outcomes in patients with advanced disease by alleviating the inhibition of effector T cells and enhancing antitumor immune responses (6). KEYNOTE-590 was the first phase III trial to demonstrate the efficacy of programmed cell death protein-1 (PD-1) inhibitor-based immunochemotherapy as a first-line treatment for advanced or metastatic esophageal squamous cell carcinoma (ESCC) and esophageal adenocarcinoma (EAC). This study evaluated pembrolizumab, a monoclonal antibody (mAb) targeting PD-1, in combination with fluorouracil and cisplatin (FP) chemotherapy, compared to chemotherapy alone, which demonstrated a significant survival benefit with the median progression-free survival (mPFS) improved from 5.8 to 6.3 months, while median overall survival (mOS) increased from 9.8 to 12.4 months in the overall population (7). The benefit was particularly pronounced in patients with programmed death receptor ligand-1 (PD-L1) combined positive score (CPS) ≥ 10 in ESCC, where mOS was extended from 8.8 months to 13.9 months (7). Subsequent trials, including CheckMate-648 (8), JUPITER-06 (9), and ASTRUM-007 (10), have further validated the broad applicability of anti-PD-1-based immunochemotherapy in advanced esophageal cancer. CheckMate-648 focused exclusively on ESCC patients and introduced the use of nivolumab (an anti-PD-1 antibody) combined with FP chemotherapy, with or without ipilimumab (an anti-CTLA-4 antibody). The study demonstrated a significant improvement in OS with nivolumab plus chemotherapy (13.2 months) and nivolumab plus ipilimumab (12.7 months) compared to chemotherapy alone (10.7 months) (8). However, the addition of ipilimumab did not confer additional benefit in terms of PFS, with nivolumab plus ipilimumab yielding a shorter median PFS (2.9 months) than both nivolumab plus chemotherapy (5.8 months) and chemotherapy alone (5.6 months) (8). These findings established anti-PD-1 plus FP chemotherapy as a preferred first-line strategy in ESCC. JUPITER-06, a trial conducted in a Chinese population, expanded the chemotherapy backbone to paclitaxel plus cisplatin (TP) and evaluated toripalimab, another anti-PD-1 antibody. It also demonstrated significant survival benefits, with median OS improved from 11 to 17 months (9). Building on these advances, the ASTRUM-007 trial, initiated in 2019, enrolled Chinese patients with PD-L1 CPS ≥ 1 ESCC, and adopted a more intensive every 2 weeks (Q2W) dosing schedule of serplulimab plus FP chemotherapy (10). This approach yielded a substantial improvement in OS (15.3 vs. 11.8 months), with acceptable toxicity (10).

Despite these advances, several important questions remain unresolved. First, although most pivotal trials enrolled PD-L1-negative patients, clinical benefit from PD-1 inhibitors has been most pronounced in patients with high PD-L1 expression (CPS ≥ 10). In particular, ASTRUM-007 only included patients with ESCC and CPS ≥ 1, leaving the efficacy of serplulimab in PD-L1-negative tumors and other histologic subtypes unexplored. Second, unlike other PD-1 inhibitors, serplulimab demonstrated anti-tumor activity in small cell lung cancer in the ASTRUM-005 trial (11), raising the hypothesis that it may offer benefit in small cell esophageal carcinoma (SCEC) as well, which is an aggressive and poorly characterized histology that has not been investigated in prior immunotherapy trials. Third, real-world treatment regimens often diverge from trial protocols. While ASTRUM-007 used FP chemotherapy administered Q2W, the TP regimen every 3 weeks (Q3W) is more commonly used in routine clinical practice in China (12). The real-world effectiveness and safety of serplulimab in this context remain unknown. Finally, randomized controlled trials (RCTs) impose strict eligibility criteria that may not reflect real-world patients, who may have comorbidities, lack PD-L1 testing due to financial or disease-related constraints, or receive individualized treatment decisions based on physician preference.

Looking beyond the current paradigm of PD-1/PD-L1 inhibition combined with chemotherapy, the future of esophageal cancer therapy is rapidly evolving toward more sophisticated and personalized strategies. An increasing number of emerging therapeutic modalities, such as antibody-drug conjugates (ADCs) and cellular therapies, are under active exploration (13, 14). However, the transition from developing novel therapies to achieving regulatory approval and widespread clinical implementation is inherently protracted and complex. Therefore, while drug development continues to advance, there remains a critical, concurrent imperative to maximize therapeutic outcomes for patients using currently available agents. Given these gaps and the imperative to inform future innovation, we conducted a national, multicenter, observational study to evaluate the real-world effectiveness and safety of first-line serplulimab-based regimens in a broader population of patients with advanced esophageal cancer, irrespective of PD-L1 expression or SCEC histologic subtype.

## 2 Methods

### 2.1 Study design and patients

This was a multicenter, observational real-world cohort study designed to evaluate the effectiveness and safety of serplulimab-based first-line treatment in patients with locally advanced or metastatic esophageal cancer across routine clinical practice in China. The study was conducted in accordance with the principles of the Declaration of Helsinki (1964, and its subsequent revisions) and followed Good Clinical Practice (GCP) guidelines where applicable to observational studies. The study also adhered to the STROBE (Strengthening the Reporting of Observational Studies in Epidemiology) guidelines for the conduct and reporting of observational cohort studies (Supplementary Table 1). The study protocol was approved by the Ethics Committee of Jiangsu Cancer Hospital & Jiangsu Institute of Cancer Research & The Affiliated Cancer Hospital of Nanjing Medical University (Approval Number: 2023-KK-083), and by the Institutional Review Boards (IRBs) of all seven other participating centers. Informed consent was waived by our Institutional Review Board because of the observational nature of our study.

Patients were enrolled between March 2022 and December 2023 at eight academic or tertiary hospitals in China. Eligible patients were identified based on predefined inclusion and exclusion criteria and were enrolled consecutively. Inclusion criteria were: (1) age ≥18 years; (2) histologically or cytologically confirmed unresectable, locally advanced, recurrent, or metastatic esophageal cancer, regardless of histologic subtype and PD-L1 expression level; (3) initiation or planned initiation of first-line serplulimab-based therapy for advanced disease; and (4) presence of at least one measurable lesion according to Response Evaluation Criteria in Solid Tumors (RECIST), version 1.1, prior to treatment. Patients were eligible if they had disease recurrence more than 6 months after completion of (neo)adjuvant chemotherapy, chemoradiotherapy, or radiotherapy. Exclusion criteria included prior participation in any clinical trial involving serplulimab and the presence of any comorbidities or conditions deemed by the investigators to interfere with study participation or assessment of outcomes.

### 2.2 Treatment and data collection

For each eligible patient, baseline clinical and demographic data were extracted from electronic medical records (EMRs). Missing or incomplete information was supplemented through telephone contact or in-person follow-up conducted by the local research team. At each participating site, two independent clinical investigators entered the data into a standardized case report form (CRF) designed for this study. Upon completion of site-level data collection, all CRFs were submitted to the central study coordinating team, who performed data validation, consistency checks, and issued data queries as necessary. Site investigators were requested to resolve any data discrepancies or missing entries through re-review of original records or patient contact. The treatment regimens, including the dosage and schedule of serplulimab, the chemotherapy backbone (e.g., paclitaxel-cisplatin or fluorouracil-cisplatin), and any concomitant targeted therapies, were selected at the discretion of the treating physicians based on individual patient characteristics and institutional practice. All treatment-related details were recorded in the CRFs. Patients were followed monthly during treatment, and clinical information, including tumor response, disease progression, and adverse events (AEs), was updated at each visit. If patients missed scheduled follow-up visits, investigators conducted telephone interviews to collect the most recent information regarding treatment continuation, clinical status, and safety outcomes, which were subsequently documented in the CRF.

### 2.3 Outcome

The primary effectiveness outcomes of this study were real-world PFS (rwPFS) and OS. Secondary endpoints included objective response rate (ORR), disease control rate (DCR), duration of response (DoR), time to first disease progression (TDP), time to treatment discontinuation (TTD), and safety. Tumor response was assessed according to the RECIST version 1.1. In brief, complete response (CR) was defined as the disappearance of all target lesions and normalization of pathologic lymph nodes (<10 mm in short axis), partial response (PR) as a ≥ 30% decrease in the sum of diameters of target lesions, progressive disease (PD) as a ≥ 20% increase in the sum of diameters from the nadir (with an absolute increase of at least 5 mm) or the appearance of new lesions, and stable disease (SD) as any response not meeting criteria for PR or PD. Given the non-interventional nature of real-world observational studies, a subset of patients declined follow-up imaging assessments. For these individuals, clinical progression was determined based on documented worsening of tumor-related symptoms and/or rising levels of tumor-associated biomarkers in peripheral blood, as judged by the treating physician. Real-world PFS was defined as the time from the initiation of first-line treatment to the date of radiographic or clinical disease progression, or death from any cause, whichever occurred first. OS was defined as the time from treatment initiation to death from any cause. ORR was calculated as the proportion of patients achieving CR or PR. DCR was defined as the proportion of patients achieving CR, PR, or SD. DoR was measured from the date of the first documented CR or PR to the date of PD, clinical progression, or death. TDP was defined as the interval from treatment initiation to the first recorded radiographic or clinical progression. TTD was defined as the time from initiation of serplulimab treatment to permanent discontinuation of any component of the regimen, or until data cutoff. Safety outcomes included the incidence and severity of AEs during the treatment, which were assessed and graded according to the Common Terminology Criteria for Adverse Events (CTCAE) version 5.0.

### 2.4 Statistical analysis

Continuous variables were summarized as means ± standard deviations (SD) for normally distributed data, or as medians with ranges or interquartile ranges (IQRs) for non-normally distributed data. Categorical variables were reported as counts and percentages (n, [%]). ORR and DCR, along with their corresponding 95% confidence intervals (CIs), were calculated using the Clopper-Pearson exact method. Time-to-event endpoints, including rwPFS, OS, DoR, TDP, and TTD, were estimated using the Kaplan–Meier method, and median survival times with 95% CIs were reported. Median follow-up time was calculated using the reverse Kaplan–Meier method. Subgroup comparisons were performed for rwPFS and OS using the log-rank test, and for ORR using the chi-squared test or Fisher’s exact test, as appropriate. Subgroup variables included age, sex, histologic subtype, ECOG performance status (PS), presence of distant metastasis, number of treatment cycles, concomitant therapies, surgical history, and initial serplulimab dose. Univariable logistic regression was used to explore associations between baseline variables and ORR, with results presented as odds ratio (OR) and corresponding 95% CIs. Univariable Cox proportional hazards models were applied to identify factors associated with rwPFS, and results were displayed as hazard ratios (HR) with corresponding 95% CIs. Variables with a *p*-value <0.20 in univariable analysis were included in multivariable regression models. All statistical analyses were performed using R software (version 4.2.3), with statistical significance defined as a two-tailed *α*-level of 0.05.

## 3 Results

### 3.1 Baseline characteristics of the patients

As of the data cutoff date (May 14th, 2024), 104 patients were included. The baseline characteristics were summarized in Table 1. The mean age of the patients was 68.65 ± 8.08 years, with 79 patients (76.0%) aged 65 years and above. The majority were male (*n* = 81, 77.9%) and had an ECOG PS of 0 (*n* = 68, 65.4%). ESCC (*n* = 84, 80.8%) was the most common histological type, followed by combined small cell and adenocarcinoma (*n* = 10, 9.6%), combined neuroendocrine and non-neuroendocrine carcinoma (*n* = 6, 5.8%), and neuroendocrine neoplasm (*n* = 4, 3.8%). Additionally, most patients did not exhibit liver metastases (*n* = 86, 82.7%).

**Table 1:** Characteristics of the patients.

Variables	All (*n* = 104)
Age (years), mean ± SD	68.65 ± 8.08
Age (years), *n* (%)	
<65	25 (24.0)
≥65	79 (76.0)
Sex, *n* (%)	
Male	81 (77.9)
Female	23 (22.1)
BMI (kg/m^2^), median (IQR)	23.84 (22.00, 25.36)
Education level, *n* (%)	
Illiteracy	45 (43.3)
Primary school	43 (41.3)
Junior high school	12 (11.5)
Technical secondary school/high school	4 (3.8)
Smoking history, *n* (%)	4 (3.8)
Alcohol use history, *n* (%)	2 (1.9)
ECOG score, *n* (%)	
0	68 (65.4)
1	36 (34.6)
Disease course (years), median (IQR)	0.03 (0.00, 0.14)
Histological type, *n* (%)	
Combined small cell and adenocarcinoma	10 (9.6)
Combined neuroendocrine and non-neuroendocrine carcinoma	6 (5.8)
Squamous cell carcinoma	84 (80.8)
Neuroendocrine neoplasm	4 (3.8)
Pathological differentiation, *n* (%)	
Highly differentiated	5 (4.8)
Moderately differentiated	31 (29.8)
Poorly differentiated	24 (23.1)
Not evaluable	44 (42.3)
Clinical stage, *n* (%)	
Cervical esophagus	2 (1.9)
Upper thoracic esophagus	17 (16.3)
Middle thoracic esophagus	49 (47.1)
Lower thoracic esophagus	29 (27.9)
Primary lesion resection	7 (6.7)
Metastasis to liver, *n* (%)	
No	86 (82.7)
Yes	18 (17.3)
T stage, *n* (%)	
T1	2 (1.9)
T2	4 (3.8)
T3	37 (35.6)
T4	8 (7.7)
Tx	53 (51.0)
N stage, *n* (%)	
N0	6 (5.8)
N1	19 (18.3)
N2	48 (46.2)
N3	7 (6.7)
Nx	24 (23.1)
M stage, *n* (%)	
M0	58 (55.8)
M1	46 (44.2)
CPS, *n* (%)	
<1	2 (1.9)
≥1	8 (7.7)
Unknown	94 (90.4)
Prior treatment history, *n* (%)	
Surgery	23 (22.1)
Radiation	5 (4.8)
Chemotherapy	8 (7.7)

SD, standard deviation; BMI, body mass index; IQR, interquartile range; ECOG, Eastern Cooperative Oncology Group; CPS, combined positive score.

### 3.2 Treatment patterns

Regarding the prior treatments, 23 patients (22.1%) underwent surgery, eight (7.7%) received chemotherapy, and five (4.8%) received radiotherapy. The median number of serplulimab treatment cycles was four (IQR: 2–6). The initial dose of serplulimab was 200 mg in 51 patients (49%), 300 mg in 52 patients (50%), and unspecified in one patient. In addition, 10 patients (9.6%) concurrently received targeted therapy, 21 (20.2%) received radiotherapy, and 97 (93.3%) received chemotherapy. The most common chemotherapy regimen was platinum plus paclitaxel (*n* = 47, 45.2%), followed by platinum plus etoposide (*n* = 15, 14.4%). Platinum-based agents (*n* = 74, 71.2%) and taxanes (nab-paclitaxel: *n* = 30, 28.8%; liposomal paclitaxel: *n* = 20, 19.2%) constituted the primary chemotherapeutic drugs.

### 3.3 Effectiveness and subgroup analysis

**Figure 1:**
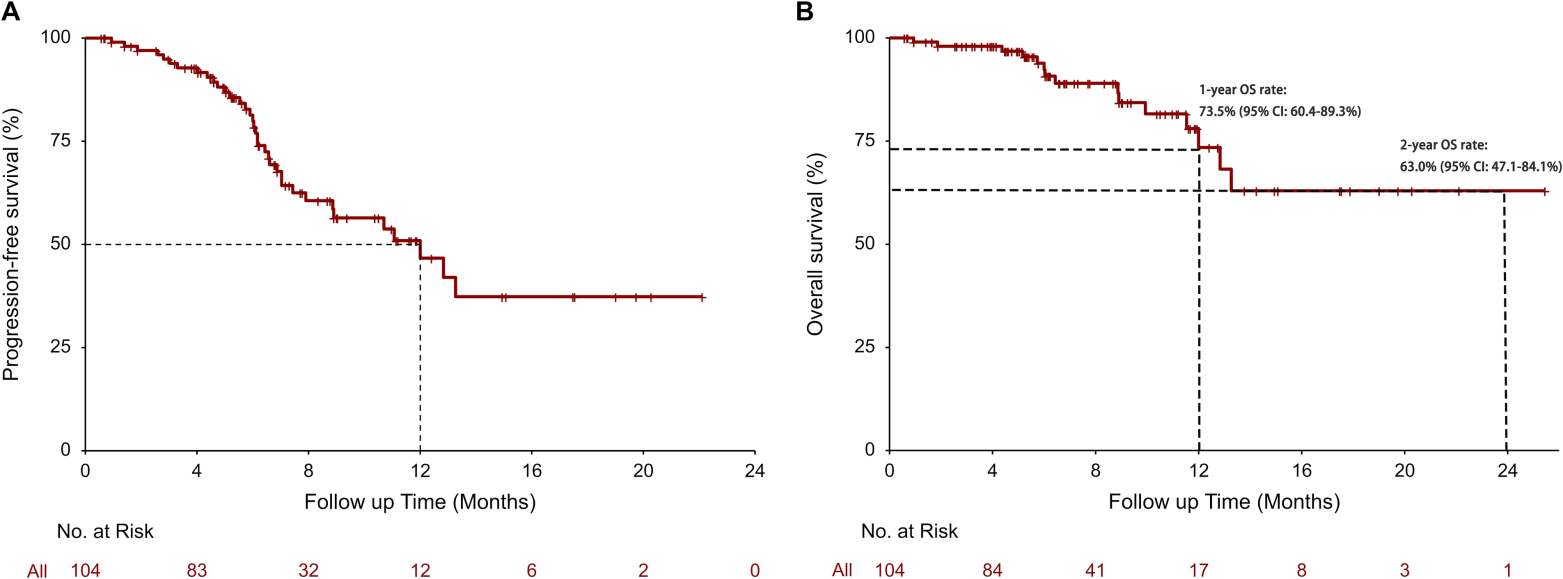
Real-world progression-free survival (rwPFS) **(A)** and real-world overall survival (rwOS) **(B)** in all patients.

The tumor response and survival outcomes for the entire cohort and subgroups are displayed in Table 2. The median follow-up duration was 6.8 months. In the survival analysis based on 36 (34.6%; radiographic progression: *n* = 17, clinical progression: *n* = 19) rwPFS events, the median rwPFS was 12.00 months (95% CI: 8.87-not reached [NR]), with a 1-year rwPFS rate of 46.7% (95% CI: 34.2–63.6%) (Figure 1A). A total of 15 patients (14.4%) reported death, and the median OS was NR (95% CI: 13.27-NR), with 1-year and 2-year OS rates of 73.5% (95% CI: 60.4–89.3%), and 63.0% (95% CI: 47.1–84.1%), respectively (Figure 1B). The confirmed ORR was 40.0% (36/90; 95% CI: 29.8–50.9%), and DCR was 97.8% (88/90; 95% CI: 92.2–99.7%). The median DoR was immature, and the median TTD and TDP were 12.00 months (95% CI: 8.87-NR) and 16.50 months (95% CI: 8.90-NR), respectively. A total of 14 patients were excluded from the ORR/DCR analysis due to clinical deterioration requiring transition to best supportive care before first evaluation (*n* = 3), non-RECIST-evaluable community-based assessments (*n* = 5), and irretrievable radiological documentation (*n* = 6).

**Table 2:** ORR and PFS.

Variables	ORR, *n* (%)	*p*	PFS, median (95% CI)	*p*
Overall	36 (40.0)		12.00 (8.87-NR)	
Age		>0.999		0.022
<65 years	8 (40.0)		6.87 (5.53-NR)	
≥65 years	28 (40.0)		12.00 (8.90-NR)	
Sex		0.605		0.594
Male	27 (38.6)		12.00 (8.87-NR)	
Female	9 (45.0)		8.90 (7.03-NR)	
ECOG score		0.117		0.797
0	21 (34.4)		12.00 (7.90-NR)	
1	15 (51.7)		8.90 (7.03-NR)	
Histological type		0.333		0.427
Squamous cell carcinoma	27 (37.5)		12.00 (10.70-NR)	
Other	9 (50.0)		7.90 (6.60-NR)	
M stage		0.730		0.734
M0	18 (38.3)		11.07 (8.87-NR)	
M1	18 (41.9)		NR (7.03-NR)	
Previous surgery		0.833		0.028
No	28 (39.4)		12.83 (8.87-NR)	
Yes	8 (42.1)		8.90 (6.03-NR)	
Number of treatment cycles		0.223		0.490
≤4	18 (34.6)		12.00 (8.87-NR)	
>4	18 (47.4)		11.07 (7.43-NR)	
Serplulimab starting dose		0.547		0.792
200 mg	20 (43.5)		12.00 (8.90-NR)	
300 mg	16 (37.2)		11.07 (6.87-NR)	
Combined with targeted therapy		0.005*		0.524
No	36 (45.0)		12.00 (7.90-NR)	
Yes	0^a^		8.90 (6.00-NR)	
Combined with platinum-based drugs		0.016		0.252
No	5 (20.0)		12.00 (6.43-NR)	
Yes	31 (47.7)		NR (8.87-NR)	
Combined with nab-paclitaxel		0.133		0.108
No	22 (34.9)		10.70 (6.87-NR)	
Yes	14 (51.9)		NR (8.90-NR)	
Combined with paclitaxel liposome		0.015		0.076
No	33 (46.5)		NR (8.87-NR)	
Yes	3 (15.8)		7.03 (6.03-NR)	
Combined with paclitaxel		0.083		0.266
No	27 (36.0)		12.00 (7.03-NR)	
Yes	9 (60.0)		NR (7.90-NR)	
Combined with etoposide		0.154		0.704
No	28 (36.8)		12.00 (8.90-NR)	
Yes	8 (57.1)		7.43 (6.10-NR)	
Combined with S-1		0.709*		0.460
No	32 (39.0)		12.83 (7.90-NR)	
Yes	4 (50.0)		8.87 (6.17-NR)	
Combined with radiotherapy		0.605		0.954
No	27 (38.6)		12.00 (7.90-NR)	
Yes	9 (45.0)		12.83 (7.03-NR)	
Metastasis to liver		0.509		0.934
No	28 (38.4)		12.00 (8.87-NR)	
Yes	8 (47.1)		7.90 (6.10-NR)	
Chemotherapy regimen		0.090*		0.295
Platinum plus paclitaxel	21 (48.8)		NR (10.70-NR)	
Platinum plus etoposide	8 (57.1)		7.43 (6.10-NR)	
Platinum plus others	2 (25.0)		8.87 (6.57-NR)	
Paclitaxel plus others	3 (17.6)		12.00 (7.03-NR)	
Other	2 (25.0)		6.00 (4.73-NR)	

*Fisher’s exact test. ^a^There was no patient who achieved a tumor response in the subgroup receiving concurrent targeted therapy (0/10, 0%). ORR, objective response rate; PFS, progression-free survival; CI, confidence interval; NR, not reached; ECOG, Eastern Cooperative Oncology Group.

**Figure 2:**
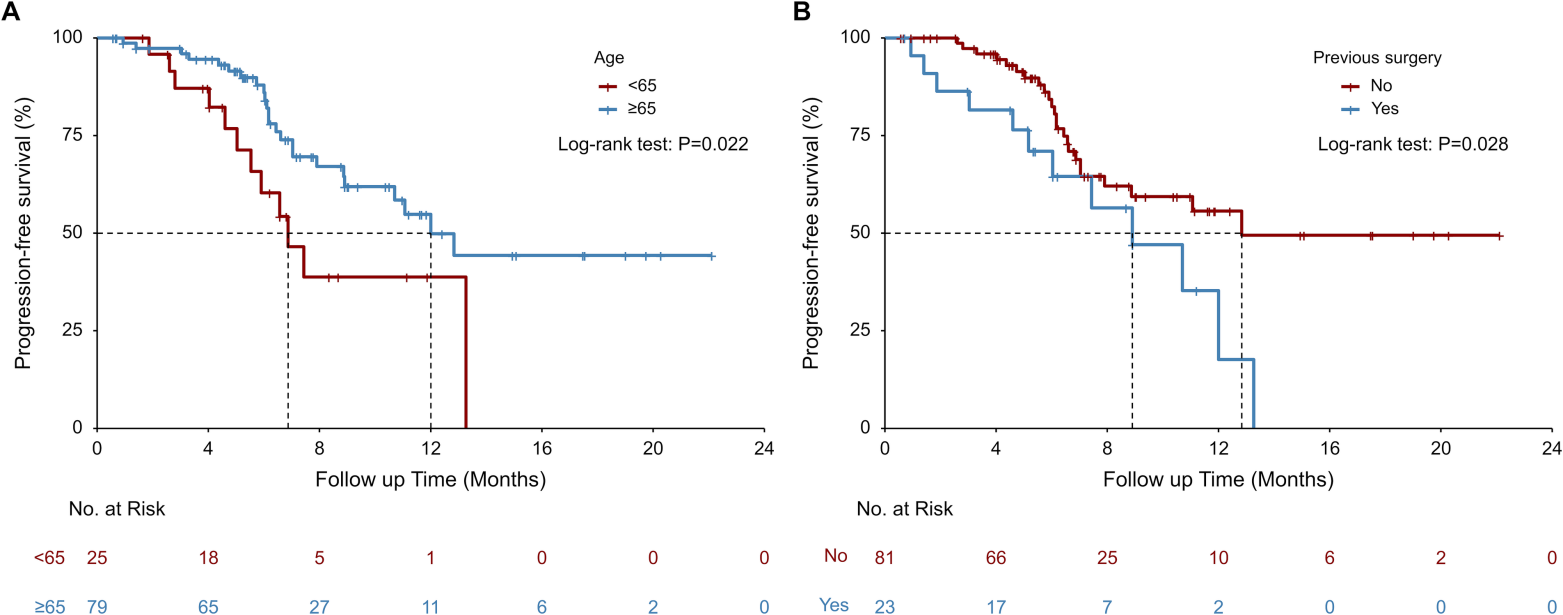
Real-world progression-free survival (rwPFS) stratified by age **(A)** and previous surgery **(B)** subgroups.

Univariate Cox model and Kaplan–Meier analysis revealed that patients aged ≥65 could achieve a significant longer median rwPFS (12.00 vs. 6.87 months, *p* = 0.022; Table 2; Figure 2A) compared to those aged <65 years following first-line serplulimab treatment, with a HR of 0.45 (95% CI: 0.22–0.91; *p* = 0.026; Table 3). Additionally, patients who underwent surgery exhibited a shorter median rwPFS than non-surgical patients (8.90 vs. 12.83 months; *p* = 0.028; Table 2; Figure 2B), associated with an increased risk of progression (HR = 2.14; 95% CI: 1.07–4.29; *p* = 0.032; Table 3), which may reflect selection bias toward surgically eligible patients who later progress or more aggressive tumor biology at baseline. On multivariate analysis, the association of rwPFS with age and surgery history was not confirmed (all *p* > 0.05), suggesting these two factors were not independently associated with the rwPFS.

**Table 3:** Univariable and multivariable analyses of ORR and PFS.

Variables	ORR				PFS			
	Univariable analysis		Multivariable analysis		Univariable analysis		Multivariable analysis	
	OR (95% CI)	*p*	OR (95% CI)	*p*	HR (95% CI)	*p*	HR (95% CI)	*p*
Age								
<65 years	1				1		1	
≥65 years	1.00 (0.36–2.76)	>0.999			0.45 (0.22–0.91)	0.026	0.56 (0.27–1.24)	0.156
Sex								
Male	1				1			
Female	1.30 (0.48–3.56)	0.605			1.23 (0.58–2.61)	0.595		
ECOG score								
0	1		1		1			
1	2.04 (0.83–5.02)	0.120	2.38 (0.81–6.97)	0.114	1.09 (0.56–2.14)	0.797		
Histological type								
Squamous cell carcinoma	1				1			
Other	1.67 (0.59–4.72)	0.336			1.34 (0.65–2.80)	0.430		
M stage								
M0	1				1			
M1	1.16 (0.50–2.70)	0.730			0.89 (0.46–1.72)	0.735		
Previous surgery								
No	1				1		1	
Yes	1.12 (0.40–3.12)	0.833			2.14 (1.07–4.29)	0.032	1.85 (0.85–4.05)	0.121
Number of treatment cycles								
≤4	1				1			
>4	1.70 (0.72–4.00)	0.224			0.79 (0.40–1.55)	0.491		
Serplulimab starting dose								
200 mg	1				1			
300 mg	0.77 (0.33–1.80)	0.547			1.09 (0.56–2.13)	0.792		
Combined with targeted therapy								
No	1				1			
Yes	0.00 (0.00-Inf)	0.989			1.36(0.53–3.51)	0.526		
Combined with platinum								
No	1		1		1			
Yes	3.65 (1.22–10.89)	0.020	2.73 (0.21–36.33)	0.447	0.68 (0.35–1.33)	0.255		
Combined with nab-paclitaxel								
No	1		1		1		1	
Yes	2.01 (0.80–5.01)	0.136	1.27 (0.18–8.82)	0.807	0.49 (0.20–1.19)	0.116	1.26 (0.31–5.15)	0.751
Combined with paclitaxel liposome								
No	1		1		1		1	
Yes	0.22 (0.06–0.81)	0.023	0.28 (0.03–2.86)	0.284	1.87 (0.93–3.78)	0.081	3.02 (0.79–11.59)	0.107
Combined with paclitaxel								
No	1		1		1			
Yes	2.67 (0.86–8.30)	0.090	2.15 (0.34–13.47)	0.415	0.56 (0.20–1.58)	0.274		
Combined with etoposide								
No	1		1		1			
Yes	2.29 (0.72–7.27)	0.161	1.09 (0.10–11.34)	0.944	1.17 (0.51–2.70)	0.705		
Combined with S-1								
No	1				1			
Yes	1.56 (0.37–6.70)	0.548			1.43 (0.55–3.70)	0.463		
Combined with radiotherapy								
No	1				1			
Yes	1.30 (0.48–3.56)	0.605			0.98 (0.47–2.06)	0.954		
Metastasis to liver								
No	1				1			
Yes	1.43 (0.50–4.14)	0.511			1.03 (0.47–2.29)	0.934		
Chemotherapy regimen								
Platinum plus paclitaxel	1		1		1		1	
Platinum plus etoposide	1.40 (0.41–4.71)	0.590	–		1.67 (0.64–4.33)	0.295	2.63 (0.64–10.78)	0.178
Platinum	0.35 (0.06–1.93)	0.227	0.29 (0.02–4.07)	0.359	2.02 (0.64–6.37)	0.231	2.85 (0.61–13.33)	0.183
Paclitaxel	0.22 (0.06–0.90)	0.034	0.92 (0.07–12.56)	0.949	1.61 (0.68–3.81)	0.279	1.01 (0.37–2.76)	0.993
Other	0.35 (0.06–1.93)	0.227	–		3.31 (1.03–10.65)	0.044	5.90 (1.25–27.89)	0.025

ORR, objective response rate; PFS, progression-free survival; OR, odds ratio; HR, hazard ratio; CI, confidence interval; ECOG, Eastern Cooperative Oncology Group.

We conducted subgroup analyses using both univariate and multivariate logistic regression models to identify the predictors for tumor response. The univariate logistic regression analysis for ORR demonstrated that treatment with platinum-based therapy (OR = 3.65; 95% CI: 1.22–10.89; *p* = 0.020) was associated with significantly higher ORR (47.7% vs. 20.0%; *p* = 0.016), whereas paclitaxel liposome (OR = 0.22; 95% CI: 0.06–0.81; *p* = 0.023) was correlated with lower ORR (15.8% vs. 46.5%; *p* = 0.015) compared to non-exposed groups (Tables 2, 3). However, the multivariable logistic regression analysis showed that no factor was independently associated with the ORR (Table 3).

### 3.4 Safety

Safety profile was assessed in all 104 patients. The incidence of AEs was 55.8% (*n* = 58), while the incidence of treatment-related adverse events (TRAEs) was 18.3% (*n* = 19), and the incidence of serious adverse events (SAEs) was 1.9% (*n* = 2, one patient with hyperkalemia and one with decreased white blood cell count). The incidence of grade ≥3AEs was 10.6% (*n* = 11). The incidence of AE leading to treatment interruption was 2.9% (*n* = 3), including two patients who developed a pulmonary infection (one with grade 1 and one with grade 3) and one patient who developed grade 3 decreased platelet count and grade 4 decreased white blood cell count. The most common AEs were hematologic toxicity, including anemia (*n* = 33, 31.7%), decreased neutrophil count (*n* = 16, 15.4%), decreased platelet count (*n* = 16, 15.4%) and decreased white blood cell count (*n* = 13, 12.5%) (Table 4).

**Table 4:** Safety.

Safety Profile	All (*n* = 104)
AEs, *n* (%)	58 (55.8)
Grade ≥3 AEs, *n* (%)	11 (10.6)
TRAE, *n* (%)	19 (18.3)
SAE, *n* (%)	2 (1.9)
AE leading to treatment discontinuation, *n* (%)	3 (2.9)
Specific AEs, *n* (%)	
Anemia	33 (31.7)
Decreased neutrophil count	16 (15.4)
Decreased platelet count	16 (15.4)
Decreased white blood cell count	13 (12.5)
Elevated lactate dehydrogenase	9 (8.7)
Elevated alanine aminotransferase	8 (7.7)
Elevated alkaline phosphatase	8 (7.7)
Hypercholesterolemia	6 (5.8)
Elevated aspartate aminotransferase	6 (5.8)
Hypoproteinemia	5 (4.8)
Infectious pneumonia	3 (2.9)
Elevated serum creatinine	3 (2.9)
Positive urine erythrocytes	2 (1.9)
Hyperlipidemia	2 (1.9)
Hyperglycemia	2 (1.9)
Elevated gamma-glutamyl transferase	2 (1.9)
Neutropenia	1 (1.0)
Hypomagnesemia	1 (1.0)
Hyponatremia	1 (1.0)
Hypothyroidism	1 (1.0)
Hypoglycemia	1 (1.0)
Hyperkalemia	1 (1.0)
Elevated blood bilirubin	1 (1.0)
Fatigue	1 (1.0)
Rash	1 (1.0)
Hypertriglyceridemia	1 (1.0)
Urinary tract infection	1 (1.0)

AE, adverse event; TRAE, treatment-related adverse event; SAE, serious adverse event.

## 4 Discussion

The landmark ASTRUM-007 trial established serplulimab as a first-line therapeutic option for advanced ESCC patients with PD-L1 CPS ≥ 1, demonstrating robust efficacy and manageable safety within a strictly controlled trial setting (10). However, generalizability to the real-world population, which encompasses heterogeneous histologic subtypes and variable PD-L1 expression levels, remains uncertain due to the inherent selection biases of RCTs. This multicenter real-world cohort study addresses this critical knowledge gap by evaluating first-line serplulimab in unselected patients with advanced esophageal cancer. Strikingly, we observed comparable tumor response signals to ASTRUM-007, with an ORR of 40.0%, and a DCR of 97.8%. Additionally, first-line serplulimab-based treatment yielded promising survival outcomes regardless of histologic type and PD-L1 expression, with a median rwPFS of 12.00 months and a 1-year rwOS rate of 73.5%. The results strongly suggested that serplulimab as a first-line treatment is effective for patients with advanced esophageal cancer, with no new safety signals observed.

The numerically attenuated ORR (40.0% vs. 57.6%) but enhanced DCR (97.8% vs. 79.6%) and prolonged rwPFS (12.00 vs. 5.8 months) observed in the present study, compared to the ASTRUM-007 trial (10), underscore a paradigm shift in therapeutic outcomes when transitioning from controlled trials to real-world complexity. The divergence may be explained by several factors. Firstly, non-ESCC subtypes, such as combined small cell and adenocarcinoma, combined neuroendocrine and non-neuroendocrine carcinoma, and neuroendocrine neoplasm, accounted for approximately 20% of patients in this real-world cohort. These non-ESCC subtypes are historically less responsive to immunotherapy, which likely diluted the ORR estimates. The extended rwPFS in this study may arise from multimodal real-world practices, such as integrating locoregional therapies (e.g., radiotherapy in 20.2% of cases) or tailored chemotherapy regimens, which are typically excluded from RCT protocols. Indeed, RCTs are intentionally designed with rigorous eligibility criteria to optimize internal validity by minimizing confounding factors as much as possible, while real-world studies inherently capture heterogeneous populations receiving relevant therapy in routine clinical practice, prioritizing generalizability (15, 16). Notably, the 1-year OS rate in this real-world cohort paralleled ASTRUM-007, suggesting that while stringent trial endpoints may underestimate immunotherapy efficacy in unselected populations, long-term survival benefits persist across diverse clinical contexts. Methodologically, the absence of protocol-mandated PD-L1 testing, a common limitation in real-world studies, precludes definitive correlations between biomarker status and response durability. Nevertheless, the consistency between our findings and prior real-world PD-1 inhibitor data in patients regardless of PD-L1 expression reinforces that immunotherapy-based regimens achieve robust disease control and survival benefits irrespective of histologic or biomarker selection (17, 18).

Identifying predictors of therapeutic response is critical for optimizing patient selection in immunotherapy. Age-specific analyses revealed that older patients (≥65 years) achieved a median rwPFS of 12 months, numerically surpassing younger counterparts and aligning with the subgroup trend of the ASTRUM-007 study (HR 0.57 for ≥65 vs. 0.61 for <61) (10). This parallel prior meta-analysis and real-world studies of ESCC immunotherapy show superior survival in elderly cohorts (19, 20), possibly attributable to age-related immune remodeling (e.g., senescence-associated secretory phenotype enhancing immunogenicity) or fewer competing causes of death. Intriguingly, patients with prior surgery exhibited reduced rwPFS, mirroring trends in prior reports (21–23), which may stem from the aggressive biology of recurrent tumors and selection bias toward surgically eligible patients who later progress (24). Furthermore, the heterogeneity of chemotherapy regimens in our cohort underscores real-world adaptability but complicates cross-trial comparisons. Additionally, while the ASTRUM-007 trial demonstrated enhanced survival with serplulimab-chemotherapy in metastatic ESCC, particularly among patients with distant metastases (HR 0.70), its subgroup analyses were underpowered for locally advanced disease due to limited sample size (10). The post-hoc analysis of the ASTRUM-007 implicating liver metastases as a negative prognostic factor (25). Although the absence of statistically significant results, our real-world data revealed similar results. Patients with M1 disease showed promising efficacy, while those who exhibited liver metastases achieved limited efficacy following first-line serplulimab therapy, which also likely reflects insufficient statistical power from our smaller population.

Consistent with previous clinical studies of serplulimab (10, 11), no new safety signals emerged in this study, and the overall safety profile was similar to the previous reports. Nevertheless, the observed numerical reduction in the AEs incidence may reflect recall bias and incomplete documentation of AEs in real-world clinical settings, which are recognized limitations of observational analyses.

Recent advances in cancer immunotherapy extend beyond PD-1/PD-L1 blockade to include novel checkpoint targets (e.g., LAG-3, TIGIT), engineered cellular therapies, and strategies to remodel the tumor microenvironment (TME)—strategies increasingly relevant to esophageal cancer (14, 26). In melanoma, circulating biomarkers such as soluble PD-L1, cytokine signatures, and lymphocyte subsets have demonstrated potential for predicting response to PD-1/PD-L1 inhibitors (27). Next-generation CAR T-cell platforms are being designed to overcome the hostile metabolic milieu of solid tumors—enhancing mitochondrial fitness or lactate resistance to maintain effector function (28). In gastric cancer, a deeper understanding of the PD-1/PD-L1 axis has spurred trials combining multiple checkpoint inhibitors and tailoring regimens based on tumor microenvironment profiling (29). Additionally, targeting the bidirectional communication between tumor cells and tumor-associated macrophages via small extracellular vesicles offers a promising route to reprogram immunosuppressive macrophages and amplify anti-tumor immunity (30). Collectively, these multidimensional advances, including spanning predictive biomarkers, next-generation cellular engineering, microenvironment-informed combination strategies, and novel immunomodulatory targets, are reinvigorating the therapeutic landscape for solid tumors like esophageal cancer by enabling more precise and potent antitumor immunity.

Given the deep cultural integration and widespread clinical utilization of Traditional Chinese Medicine (TCM) in China, its role as a complementary approach in cancer management warrants acknowledgment. Contemporary studies indicate that certain TCM compounds exert antitumor effects through modulation of ion channels and immune pathways, though their mechanisms require further scientific validation (31–33). Notably, emerging research demonstrates the feasibility of integrating TCM with biomarker-driven strategies, as evidenced by the synergy between traditional formulations and molecular targeting in counteracting esophageal cancer progression (34). While our real-world study focuses on conventional immunotherapies, these observations highlight the potential for future investigations exploring TCM-modulated immune microenvironment remodeling as an adjunct to standard esophageal cancer regimens.

The present study had several limitations. Firstly, the real-world observational design fundamentally restricts the ability to make causal inferences between therapeutic interventions and clinical outcomes. The predominance of ESCC in our cohort and the limited number of non-ESCC cases highlight the need for larger, prospective studies specifically powered to evaluate efficacy in these rarer subtypes. Second, inherent to its observational design, critical biomarker data (e.g., PD-L1 expression levels) were unavailable, precluding correlative analyses with clinical efficacy. Most patients included in this real-world cohort did not have the PD-L1 expression level, mainly due to the financial constraints (including insurance coverage and high out-of-pocket costs for patients), insufficient tissue samples for ancillary biomarker testing after primary diagnostic procedures, and fragmentation of biomarker data across disconnected electronic health record systems. Of note, the reporting of AE may introduce potential recall bias and incomplete documentation, given the observational, real-world nature of our study, underscoring the necessity for enhanced real-time monitoring and standardized reporting in future studies in the real-world research. While real-world chemotherapy regimens are personalized for individual patients, and our study incorporated comprehensive subgroup analyses of these regimens, the clinical implications of chemotherapy heterogeneity warrant further investigation. Furthermore, while preliminary findings provide insights into therapeutic potential, prospective studies with protocol-driven biomarker assessments are warranted to conclusively establish efficacy, safety, and optimal patient selection criteria. These investigations should prioritize extended follow-up periods, mandated biomarker testing, and standardized data collection protocols to address the constraints observed in real-world observational analyses.

In conclusion, this multicenter real-world study substantiates the clinically meaningful efficacy and manageable safety profile of first-line serplulimab in advanced esophageal cancer, reinforcing its therapeutic value in real-world routine practice. Critically, while squamous histology predominated—reflecting epidemiological patterns—comparative analysis revealed comparable therapeutic outcomes between ESCC and non-ESCC subtypes, supporting serplulimab’s utility across histological spectra. Although constrained by the real-world study nature and heterogeneous treatment protocols limiting biomarker-driven analyses, the observed survival benefits harmonize with contemporary evidence supporting PD-1 inhibitor-based regimens in this population. Future prospective investigations integrating comprehensive biomarker profiling are warranted to refine patient selection criteria and elucidate mechanisms underlying interindividual response variability, particularly in subgroups with high-risk features. These efforts will advance precision immunotherapy strategies for esophageal malignancies.

## Data Availability

The original contributions presented in the study are included in the article/Supplementary material, further inquiries can be directed to the corresponding authors.
